# Transcriptomic Analysis Reveals the Involvement of lncRNA–miRNA–mRNA Networks in Hair Follicle Induction in Aohan Fine Wool Sheep Skin

**DOI:** 10.3389/fgene.2020.00590

**Published:** 2020-06-09

**Authors:** Ranran Zhao, Jing Li, Nan Liu, Hegang Li, Lirong Liu, Feng Yang, Lanlan Li, Yuan Wang, Jianning He

**Affiliations:** ^1^College of Animal Science and Technology, Qingdao Agricultural University, Qingdao, China; ^2^Qufu Animal Husbandry and Veterinary Technical Service Center, Qufu, China; ^3^China Animal Health and Epidemiology Center, Qingdao, China; ^4^College of Animal Science, Inner Mongolia Agricultural University, Hohhot, China

**Keywords:** Aohan fine wool sheep, long non-coding RNA, microRNA, mRNA, wool follicle, skin development

## Abstract

Long non-coding RNAs (lncRNA) and microRNAs (miRNA) are new found classes of non-coding RNAs (ncRNAs) that are not translated into proteins but regulate various cellular and biological processes. In this study, we conducted a transcriptomic analysis of ncRNA and mRNA expression in Aohan fine wool sheep (AFWS) at different growth stages (embryonic day 90, embryonic day 120, and the day of birth), and explored their relationship with wool follicle growth. In total, 461 lncRNAs, 106 miRNAs, and 1,009 mRNAs were found to be differentially expressed during the three stages of wool follicle development. Gene Ontology (GO) enrichment and Kyoto Encyclopedia of Genes and Genomes (KEGG) pathway analysis were performed to clarify the roles of the differentially expressed lncRNA, miRNA, and mRNA in the different stages of wool follicle development. Quantitative real-time PCR (qRT-PCR) was used to validate the results of RNA-seq analysis. lncRNA (MSTRG.223165) was found to act as a competing endogenous RNA (ceRNA) and may participate in wool follicle development by acting as an miR-21 sponge. Network prediction implicated the MSTRG.223165-miR-21-*SOX6* axis in the wool follicle development. The targeting relationships of miR-21 with *SOX6* and MSTRG.223165 were validated in dual-luciferase assays. This is the first report indicating the association of the lncRNA–miRNA–mRNA network with wool follicle development in AFWS. This study provides new insights into the regulation of the wool follicle growth and represents a solid foundation for wool sheep breeding programs.

## Introduction

The quality and yield of mammalian hair is determined by the characteristics and structure of hair follicles (HF) (Mann, 1968; Nixon et al., 1996). The Aohan fine wool sheep (AFWS) is an ancient representative of this breed-type that originated in Northeast of China. This breed is characterized by stable genetic performance, strong adaptability, and is suitable for breeding in arid areas. The wool has good textile technology performance, with a wool fineness of approximately 22 μm, and a hair length exceeding 9 cm (Wang et al., 1996). It is an excellent raw material for manufacturing fine textiles and has high economic value. The morphological structure and development of HF have important effects on wool properties. HFs are complex structures that are classified as primary hair follicles (PF) and secondary hair follicles (SF) depending on the structural characteristics and developmental stage. Compared with the SF, the diameter of the PF and hair bulbs are larger, and both have complete sebaceous glands. The SF develops later than the PF, and the diameter of the hair follicles and hair bulbs are smaller, with only a few SF having a single sebaceous gland (Carter and Hardy, 1943; Schinckel, 1953). In fine wool sheep, SFs determine fine wool production and have an important effect on fiber diameter.

In sheep breeding, wool length and fineness combined with other indicators are important standards for measuring wool quality in the textile industry. Studies of the genes that regulate HF development have become a focus of research aimed at improving wool quality and increasing wool production (Cui et al., 2010; Hamanaka et al., 2013). Studies on the growth and characteristics of sheep HF have been reported since the 1950s, and HF development is well-understood at the cellular level (Hardy and Lyne, 1956; Moore et al., 1998). Studying the molecular mechanisms that regulate the morphogenesis of secondary wool follicles in AFWS, and finding regulatory factors and functional genes that specifically regulate their growth and development will provide a reliable scientific basis for improving wool quality using molecular breeding techniques.

RNA-sequencing (RNA-seq) has been widely used to evaluate in gene expression patterns in different species or different stages of growth in the same species. This approach has been used in combination with disciplines such as differential gene expression analysis, new transcript prediction, untranslated region (UTR) analysis, alternative splicing analysis, Simple Short Repeat (SSR)/Single Nucleotide Polymorphism (SNP) analysis and non-coding RNA (ncRNA) analysis (Schliebner et al., 2014; Thakur and Randhawa, 2018). NcRNAs include transfer RNA (tRNA), microRNA (miRNA), circular RNA (circRNA), and long non-coding RNA (lncRNA). These ncRNAs cannot be translated into protein, but regulate gene expression, cell function and biological processes at the level of transcription and post transcription (Huttenhofer et al., 2005). lncRNAs represent a class of RNA molecules longer than 200 nucleotides (nt) that was first discovered by high-throughput sequencing of mouse full-length cDNA (Okazaki et al., 2002; Mercer and Mattick, 2013). miRNAs are short (19–25 nt), single-stranded ncRNAs that regulate the expression of target genes at the post-transcriptional level by partially binding to the 3′-UTR region of target mRNA, thereby playing an important role in many biological activities (Ambros, 2004; Bartel, 2004). Since the initial discovery in *C. elegans* (Lee et al., 1993), 38,589 miRNAs have been identified to date^1^. CircRNAs exist as a covalently closed loop structure, without the 5′-end cap structure or 3′-poly(A) tail (Li et al., 2018). Some circRNAs such as circZNF609 (Pamudurti et al., 2017) and circ-FBXW7 (Legnini et al., 2017) can be translated into proteins.

In recent years, scientists have discovered a new regulatory mechanism that involves competing endogenous RNA (ceRNA). Two types of RNA transcripts regulate their expression by competitively combining with miRNAs, forming a large-scale miRNA-based transcriptional regulatory network. In this study, lncRNA was found to competitively bind miRNA with mRNA via this regulatory mechanism, thereby regulating the expression of miRNA target genes after transcription.

RNA-sequencing (RNA-seq) has been used in to study a wide variety of processes including sheep reproduction (Legnini et al., 2017), sheep muscle growth (Cao et al., 2018), and liver function (Li et al., 2019). Using this approach, it was shown thatmiR-27b promotes the proliferation of sheep skeletal muscle by targeting the myostatin gene (Zhang et al., 2018). Lnc-SEMT promotes sheep myoblast differentiation *in vitro* and increases the number of muscle fibers (Wei et al., 2018). Many circRNAs have been identified in the sheep pituitary, and screening of the differentially expressed circRNAs in the prenatal and postnatal pituitary has provided an improved understanding of the function of circRNAs in the pituitary (Li et al., 2017). Studies have also identified mRNAs and lncRNAs in the uterus of multi-tailed and single-tailed small-tailed sheep (*Ovis aries*) and RNAs such as MSTRG.134747 and *UGT1A1* may play a role in the molecular mechanism of sheep fertility (La et al., 2019). However, there are few reports about the mechanism underlying regulation of the lncRNA–miRNA–mRNA network in wool sheep.

In the present study, we evaluated the histological changes in skin tissues at embryonic day 90 (E90d), at embryonic day 120 (E120d), and birth (Birth) to clarify the morphological changes of wool follicles in different stages of growth. To investigate the function of ceRNA regulation in skin wool follicle development, we explored the expression profiles of lncRNA, mRNA, and miRNA in sheep by RNA-seq. The interactions between miRNAs, mRNAs, and lncRNAs that were differentially expressed in three growth stages were determined. We constructed ceRNA regulatory networks and discovered some interesting ceRNA interactions during three stages of hair follicle development, revealing potential regulatory mechanisms, and providing the foundation for further studies on the development of AFWS wool follicles.

## Materials and Methods

### Sheep Selection and Skin Tissue Preparation

Aohan fine wool sheep is a breed of sheep farmed in China for its excellent wool and meat and strong adaptability. The experimental sheep were raised in the AFWS stud farm of Inner Mongolia Autonomous Region, and fed according to the farm’s feeding plan. Nine healthy Aohan fine wool ewes (aged 3–5 years) were fed under the same conditions, subjected to estrus treatment during September, and artificially fertilized from the same ram. On embryonic day 90 (E90d) and embryonic day 120 (E120d) after fertilization, and the day of birth (Birth), samples of shoulder skin tissue with (diameter 2 cm) were collected from nine sheep (three samples at each stage).

The ewes and lambs were anesthetized with sodium pentobarbital (25 mg/kg) by intravenous injection. After sample collection, the ewes and newborn lambs were released, whereas the fetuses from E90d and E120d were placed, still under anesthesia, inside a closed chamber, and sacrificed by carbon dioxide inhalation. The anesthesia procedure was performed following published protocols (Bennett, 2005). Samples were immediately placed in liquid nitrogen for RNA-Seq and qRT-PCR analysis.

To study the morphological changes of primary and secondary hair follicles in the three stages of development, we collected shoulder fur samples into 4% paraformaldehyde, and prepared paraffin-embedded sections that were stained with hematoxylin–eosin (HE) for histological evaluation.

### RNA Isolation and Library Construction

Total RNA was isolated from skin samples used TRIzol (Life Technologies, CA, United States). The RNA samples were evaluated for degradation and impurities by 1% agarose electrophoresis. An Agilent 2100 Bioanalyzer and Agilent RNA 6000 Nano Kit were used to assess RNA concentration and integrity (Agilent Technologies, CA, United States), and purity was determined using a NanoDrop 2000 (NanoDrop Technologies, Wilmington, DE, United States). The samples were sequenced and samples with RNA Integrity Number (RIN) scores >7 were used in subsequent analyses. The sheep genome oar_v4.0 was selected as the reference genome in this study. High -throughput sequencing was performed by Annoroad technologies (Beijing, China).

### lncRNA Library Construction and Sequencing

The lncRNA library was constructed from total RNA (3 μg) using different index tags with the super directional RNA library prep kit (NEB, Ipswich, United States) according to the manufacturer’s instructions. First, the ribosomal rRNA was removed using a kit, and the RNA fragment was transformed into a short fragment. Short sequence RNA was used as template and six base random primers (random hexamers) were to synthesize the first-strand of cDNA. The second strand of cDNA was then synthesized. Following purification with a QIA Quick PCR kit, eluted with EB buffer, followed by end-repair and the add base (A) and a sequencing connector, the target fragments were recovered by agarose gel electrophoresis. The double-stranded cDNA was digested by uracil-DNA glycosylase and amplified by PCR. Finally, the target fragments of the complete library were recovered by agarose gel electrophoresis.

### miRNA Library Construction and Sequencing

Total RNA samples were digested to generate fragments of 18–30 nt or 15–35 nt RNA and collected by agarose gel electrophoresis; the ends of the isolated RNA fragments were ligated and then reverse-transcribed into cDNA, then PCR amplification was performed to establish a sequencing library. The qualified sequencing libraries were sequenced using the Illumina platform (Illumina X Ten) with the SE50sequencing strategy.

### Identification of lncRNAs

#### Quality Control and Transcriptome Assembly

Illumina raw reads results were stored in FASTQ (fq). The original sequence contained the sequencing linker sequences and low quality sequences. To ensure the quality of the information analysis data, we filtered the original offline sequence data to obtain high quality clean reads for subsequent analysis. Data quality control involved removal of contaminated reads, low quality reads, reads containing N ratios >5%, and reads that matched the ribosomal RNA. The RNA-seq data filtered for each sample was compared with the genome by HiSAT2 (Kim et al., 2015). Fragments per kilobase million (FPKM) was used to estimate gene expression levels quantitatively according to the following formula: FPKM=$\frac{10^{3,*}\,F}{N\,L/10^{6}}$, where *F* is the number of fragments covered by each gene, *N* is the sequence of the total alignment, and *L* is the length of the gene. The FPKM method eliminates the effects of gene length and sequencing differences on gene expression.

#### lncRNA Coding Potential Prediction and Target Gene Prediction

lncRNA is a long-chain ncRNA (>200 bp) classified as lincRNA (intergenic RNA), intronic lncRNA, anti-sense lncRNA, sense lncRNA, and bidirectional lncRNA according to the positional relationship with the coding sequence. When conducting novel lncRNA screening, the three basic criteria were applied:(1) Transcript length ≥200 bp and number of exons is ≥2; (2) Coverage for each transcript was calculated and transcripts with <5 were screened out in all samples; and (3) Known mRNAs and other ncRNAs were screened. Following this initial screening of lncRNA information, four analytical methods were used to predict its coding potential: Coding-Non-Coding Index (CNCI) analysis (Sun et al., 2013), Coding Potential Calculator (CPC) analysis (Lei et al., 2007), PFAM protein domain analysis (Bateman et al., 2002; Mistry et al., 2007), and Coding Potential Assessment Tool (CPAT) analysis (Wang et al., 2013). The results predicted by the four software were used as the subsequent lncRNA analysis data set.

### Identification of miRNAs

The raw data for miRNA were stored in FASTQ (fq) format. To ensure the accuracy of our subsequent analysis, the clean reads were first mapped to the reference genome (oar_v4.0) using Bowtie software (V1.1.2) (Langmead et al., 2009), and the number of total clean reads and their alignment ratios were compared with the sequence of the specified species in the miRBase database (Release 21). The sequences matching different regions in each sample were obtained, and the expression level, sequence, length and features such as secondary structure of the known miRNAs were identified. The clean reads, which were not annotated as known miRNAs, were compared to the ncRNA sequences in Rfam (13.0) (Burge et al., 2013) to enable annotation of rRNA, tRNA, snRNA, snoRNA, and other ncRNAs.

### Identification of Differentially Expressed RNAs

Three AFWS were selected as biological replicates, to identify differentially expressed lncRNAs, mRNAs, and miRNAs. The Fragments per Kilobase per Million Mapped Fragments (FPKM) values (Trapnell et al., 2010) were used to estimate the expression of lncRNA and mRNA, and Transcripts Per Million (TPM) values (Zhou et al., 2010) were used to assess miRNA expression. Differential expression analysis of lncRNA, mRNA and miRNA was performed using DEseq (Anders and Huber, 2010). The treatment group was compared with the reference group. For lncRNA and mRNA, log2Ratio| ≥ 1 and *q* < 0.05 were used as screening conditions for significant DE genes. For miRNA, pval ≤ 0.05, padj ≤ 0.05 and | log2(Fold_change)| ≥ 1 are screening conditions for DE miRNAs.

### Quantitative Real-Time PCR

We used the CFX96 Real-Time System (BioRad, CA, United States) to validate the expression levels of DE lncRNAs, DE mRNAs, and DE miRNAs. The primer sequences of the selected lncRNAs, mRNAs and miRNAs are listed in Supplementary Table S1. For lncRNA and mRNA, total RNA was synthesized used as a template to synthesize cDNA using Transcriptor First-Strand cDNA Synthesis Kit (Roche, Australia). For miRNA, the reverse primer of the miRNAs was provided in the Mir-X^TM^ miRNA first-Strand Synthesis Kit (TaKaRa, Dalian, China) and cDNA was synthesized. The housekeeping gene GAPDH was used to normalize the expression levels of the lncRNAs and mRNAs, and U6 was used to normalize the expression levels of miRNAs. Quantitative real-time PCR was carried out in a 20-μL reaction mixture containing 10 μL 2 × iTaq^TM^ Universal SYBR^@^ Green Supermix (BioRad, CA, United States), 1 μL cDNA, 8 μL ddH_2_O, and 0.5 μL forward and reverse primers. The following thermocycling program was used: 95°C for 10 min; 45 cycles of 95°C for 10 s, 60°C for 10 s, and 72°C for 10 s; 72°C for 6 min. At least three samples were analyzed for each developmental stage (E90d, E120d, Birth) and each sample was analyzed in three independent reactions. The relative expression levels were calculated using the 2^–ΔΔCt^ method.

### Target Gene Prediction, GO Annotation, and KEGG Analysis

The DE lncRNA was subjected to *cis* and *trans* target analysis, and its function was indirectly predicted by the target gene. The principle of *cis* target gene prediction is that the function of lncRNA is related to the protein coding gene adjacent to the genomic locus, and the protein coding gene of the adjacent position (50 Kb upstream and downstream) of the lncRNA is screened as a *cis* target gene. The principle of *trans* target gene prediction is that the function of lncRNA is related to a co-expressed gene, and the *trans* target gene is screened according to the correlation coefficient of lncRNA and mRNA expression level (correlation coefficient corr ≥ 0.9).

#### miRNA Family Analysis and Target Gene Prediction

miRNA conservation is manifested as the existence of miRNAs of the same family in many closely related species. To identify the phenomenon of conservation in each species, miRNA family analysis is performed using the miRBase reference for known miRNAs. Three target prediction software packages, PITA, miRanda (Enright et al., 2003), and Target Scan, were used to predict miRNA target genes, retaining at least the target genes present in two prediction software.

Gene Ontology enrichment and KEGG pathway analysis of DE mRNA and DE lncRNA and DE miRNA target genes were performed to investigate their biological functions. GO functional analysis were used Blast2GO (Conesa et al., 2005) and KOBAS were used for GO enrichment and KEGG pathway analysis. In GO enrichment, genes are annotated according to three ontologies: molecular function (MF), the cellular component (CC), and biological process (BP). KEGG pathway analysis was then conducted to explore the significantly enriched pathways of the genes. In GO terms and KEGG pathways, *q* < 0.05 was considered to indicate a term and pathway for significant enrichment of mRNA.

### Construction of ncRNAs Regulatory Networks

MiRanda, PITA and Target Scan software were used to predict the mRNA and lncRNA targets of miRNAs. The miRNA–mRNA, lncRNA–miRNA, and lncRNA–miRNA–mRNA networks were constructed according to the predicted miRNA–mRNA and miRNA–lncRNA pairs using Cytoscape software (Shannon et al., 2003). Predicted miRNA–mRNA and miRNA–lncRNA regulatory pairs were based on differential expression during the three developmental periods. CeRNA networks were constructed based on the DE mRNAs and DE lncRNAs with the same miRNA binding sites.

### Dual-Luciferase Assay

Dual-luciferase reported assays were used to verify the predicted *SOX6* and MSTRG.223165 as targets of miR-21. We cloned the wild-type (WT) and mutant 3′-UTRs of the *SOX6* and MSTRG.223165 mRNAs containing the miR-21 target site into the psiCHECK-2 reporter plasmid to generate psiCHECK-2-SOX6-3′UTR WT, psiCHECK-2-SOX6-3′UTR-mutant, psiCHECK-2-MSTRG.223165-3′UTR WT and psiCHECK-2-MSTRG.223165-3′UTR-mutant. These constructs were then cotransfected into 293T cells with miR-21 mimics or negative control respectively, and luciferase activity was measured 48 h later. The transfected cells were disrupted by the addition of 100 μl passive lysis buffer. A sample (20 μl) of the lysis mix was then added to 100 μl Luciferase Assay Reagent II (LAR II). The Firefly luciferase activity was determined as the internal control and 100 μl Stop & Glo^®^ Reagent (Luciferase Assay Reagent, Promega) was added to determine the Renilla luciferase activity of the reporter gene.

## Results

### Hair Follicle Growth Process

**FIGURE 1:**
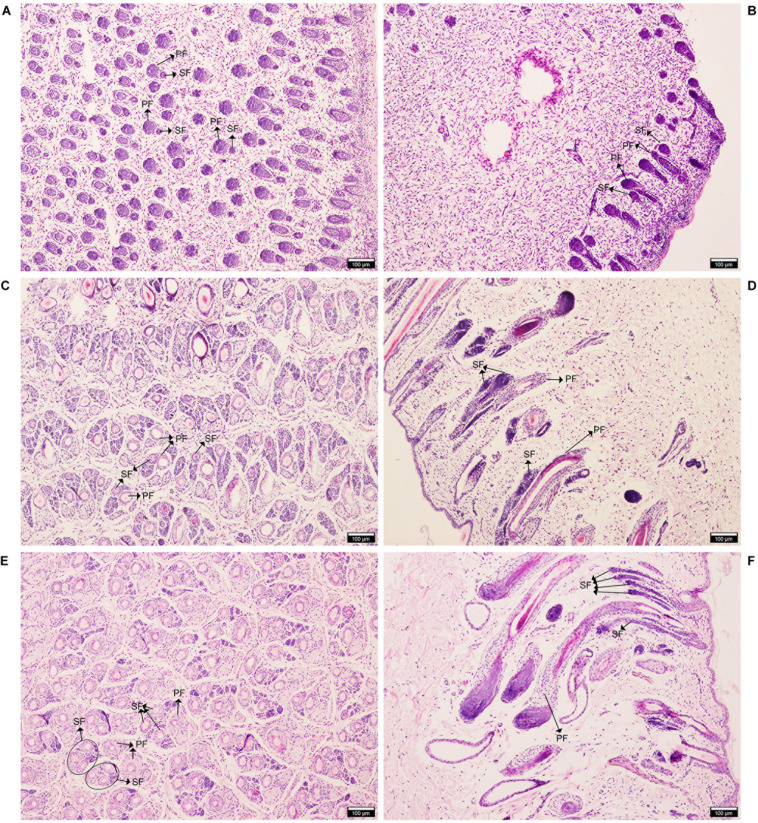
Hematoxylin and eosin (HE) staining of AFWS wool follicles at three developmental stages. Horizontal and longitudinal sections of primary and secondary wool follicles. **(A,B)** E90d, **(C,D)** E120d, **(E,F)** At birth. PF, primary wool follicle; SF, secondary wool follicle.

The HE staining showed that at E90d, most of the PF had already developed, and some of the earlier-developed PF had visible columnar structures. SF were observed around some of the PF. PF occur earlier, with larger and longer hair follicles, and accessory structures such as sweat glands, sebaceous glands, and the arrector pili muscles. SF are smaller and grow near the epidermis of the PF (Figures 1A,B). At E120d, the SF has increased in number, and were separated from the PF but arranged in parallel (Figures 1C,D). At birth, some SF mature and penetrate the body surface (Figures 1E,F). The results of this study were consistent with those of Roger (Rogers, 2006) and Liu (Liu et al., 2015), indicating that these three stages are typical periods of SF development; these results support the accuracy and representativeness of the sample collection.

### Identification of lncRNAs in AFWS Skin

**FIGURE 2:**
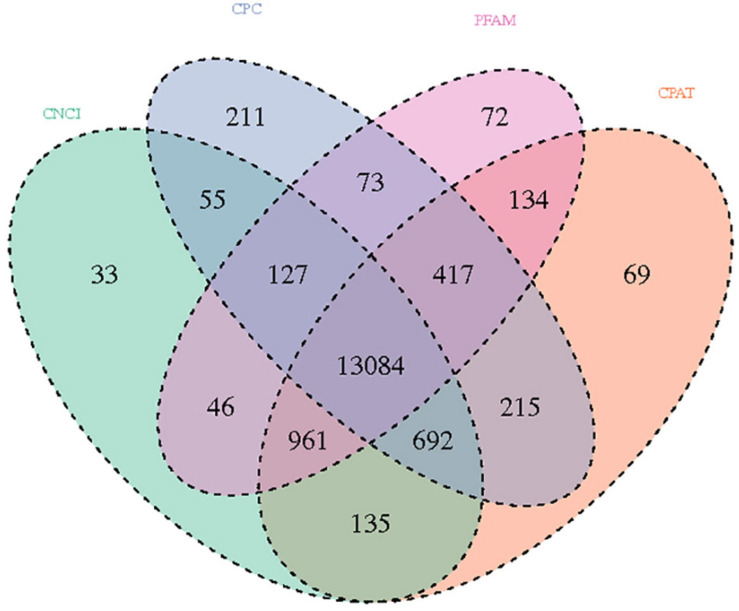
Venn diagram showing the total and unique lncRNA numbers predicted by CNCI, CPC, FPAM, and CPAT.

In the analysis results of lncRNA, a total of 895,800,158 raw reads were produced from the Illumina PE 150 platform. After the raw data was filtered, 832,283,504 clean reads were obtained for subsequent analysis. We screened novel lncRNA for three types: lincRNA, intronic lncRNA, and anti-sense lncRNA. Subsequently, CNCI analysis (Sun et al., 2013), CPC analysis (Lei et al., 2007), PFAM protein domain analysis (Bateman et al., 2002; Mistry et al., 2007) and CPAT analysis (Wang et al., 2013) were conducted to determine the coding potential of the selected novel lncRNAs; the non-coding transcripts identified using these methods are shown in Figure 2. In total, 13,148 lncRNAs predicted using these four prediction methods were selected for subsequent analysis.

### Comparative Analysis of lncRNA and mRNA

Transcription length, exon number, expression level and tissue specificity were used to compare the differences and characteristics of lncRNA and mRNA. Length distribution analysis that lncRNA was longer than mRNA (Supplementary Figure S1B), while the number and expression level of mRNAs were higher than those of lncRNAs (Supplementary Figures S1A,C). The Jensen–Shannon divergence (JS) score is an indicator of tissue expression bias, with higher scores indicating the more uneven gene expression in tissues. The JS score showed that the tissue specificity of mRNA was stronger than that of lncRNA (Supplementary Figure S1D).

The prediction of *cis* and *trans* target genes for differentially expressed lncRNAs was used to understand the function of the lncRNAs in the different growth stages. In the Birth_E90d comparison, 50 potential *cis* targets genes were identified for 26 lncRNAs, while 578 potential *trans* target genes were identified for 32 lncRNAs. In the Birth_E120d comparison, 155 potential *cis* targets genes were identified for 78 lncRNAs, while 1269 potential *trans* target genes were identified for 101 lncRNAs. In theE120d_E90d comparison, 424 potential targets *cis* genes were identified for 224 lncRNAs, while 2,256 potential *trans* target genes were identified for 235 lncRNAs. We also found that some lncRNAs have more than 50 target genes; such as MSTRG.421324, which predominantly target genes in the KRT family, according to previous reports, the KRT family is closely related to goat hair follicle growth (Gao et al., 2016), and dysregulation of *KRT* can cause hair disorders (Donet et al., 2008). LOC106990846 has 207 *trans* target genes, including *TCF12*, *VEGFA*, *FOX13*, and *SMAD* family genes (Potter et al., 2006; Kandyba et al., 2014; Du et al., 2018; Zhang et al., 2019b), all of which are involved in regulating the growth and differentiation of hair follicles. In addition, MSTRG.447490 and gene24735 target *PRR9*, which plays a regulatory role in HF keratinization and hair shaft differentiation (Gao et al., 2016). Detailed interactions between lncRNAs and mRNAs are listed in Supplementary Table S2.

### Identification of miRNAs in AFWS Skin

From the RNA-seq results, 152 known miRNAs from 77 precursors were identified (Supplementary Table S3). By comparing the sequence of the species specified in the miRBase database (Release 21), we obtained the sequences of 432 novel miRNAs and their precursor sequences (Supplementary Table S4).

### Identification of DE lncRNAs, mRNAs, and miRNAs

We used DEseq (Anders and Huber, 2010) to analyze the DE lncRNA, DE mRNA and DE miRNA according to the following screening criteria: DE lncRNA and DE mRNA, | log2Ratio| ≥ 1, and *q* < 0.05 and DE miRNA, *P* < 0.05 and | log2(Fold_change)| ≥ 1. Volcano plots, clustering maps, and Venn diagrams were used to describe the distribution of the DE lncRNAs, DE mRNAs, and DE miRNA among the three groups (Supplementary Figures S2–S4). The numbers of DE lncRNAs, DE mRNAs, and DE miRNA are listed in Table 1. A total of 41 DE lncRNAs were identified in the Birth_E90d comparison (upregulated: 35, downregulated: 6), 144 in the Birth_E120d comparison (upregulated: 125, downregulated: 19), and 384 in the E120d_E90d (upregulated: 144, downregulated: 240). A total 54 DE mRNAs were identified in the Birth_E90d (upregulated: 18, downregulated: 36), 340 in the Birth_E120d comparison (upregulated: 259, downregulated: 81), and 950 in the E120d_E90d comparison (upregulated: 306, downregulated: 644). A total 18 DE lncRNAs were identified in the Birth_E90d comparison (upregulated: 8, downregulated: 10), 31 in the Birth_E120d comparison (upregulated: 28, downregulated: 3), and 95 in the E120d_E90d comparison (upregulated: 14, downregulated: 81).

**TABLE 1:** Differentially expressed lncRNA, mRNA, and miRNA.

**Group Type**	**Regulation**	**Birth_E90d**	**Birth_E120d**	**E120d_E90d**
lncRNA	Up	35	125	144
	Down	6	19	240
	Total	41	144	384
	Up	18	259	306
mRNA	Down	36	81	644
	Total	54	340	950
	Up	8	28	14
miRNA	Down	10	3	81
	Total	18	31	95

Details of the differentially expressed lncRNAs, mRNAs and miRNA among the three developmental stages are listed in Supplementary Tables S5–S7. It was found that novel lncRNAs, such as MSTRG.617489, MSTRG.136252, MSTRG.571405, and MSTRG.223165, and known lncRNAs, such as, LOC105613955, LOC105616569, and LOC105606646, were significantly differentially expressed and had high FPKM, indicating that these lncRNAs play a role during the growth of hair follicles. Moreover, several mRNAs, such as *VCAN*, *KRT*, *HOXC13*, and *PRR9* as well as miRNAs such as the miR-200 family, let-7 family, miR-148a, and miR-143, detected in this study, which may have important roles in HF growth and development and were therefore implicated in these processes in AFWS.

### Validation of RNA-Seq Results by qRT-PCR

**FIGURE 3:**
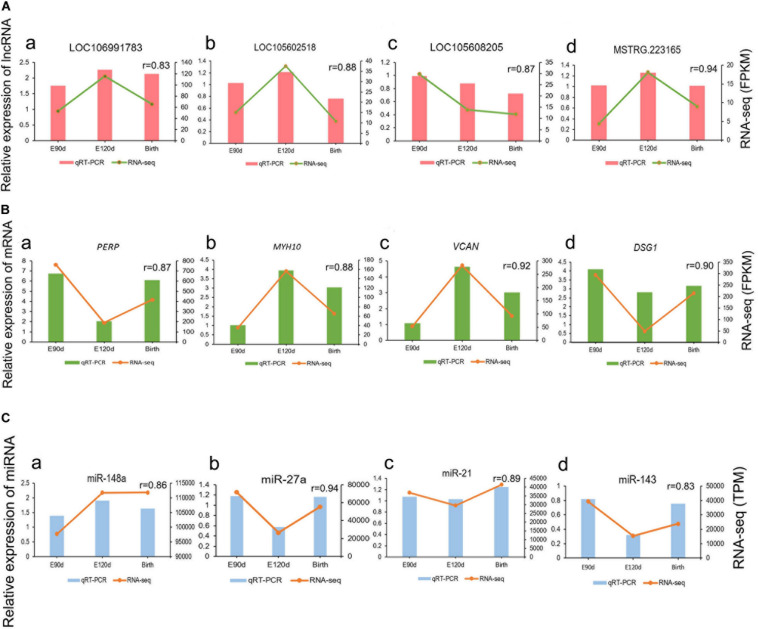
Validation of differentially expressed lncRNAs **(A)**, mRNAs **(B)**, and miRNAs **(C)** at the E90d, E120d, and Birth stages by qRT-PCR.

To validate the RNA-seq results, we randomly selected four highly expressed lncRNAs, four mRNAs, and four miRNAs and detected their expression levels in the E90d, E120d, and Birth samples by qRT-PCR, the results were in accordance with the RNA-seq data, the correlation results for all RNAs were *r* > 0.8, which demonstrated the high reliability of the RNA-seq data (Figure 3).

### GO Enrichment and KEGG Pathway Analysis

lncRNA function is related to the adjacent protein coding gene at the genomic locus; therefore, the 50-kb protein coding genes upstream and downstream of the lncRNA were screened for GO and KEGG enrichment. Several GO items were found to be significantly enriched in the experimental groups (Supplementary Table S8). Some GO terms were not significantly enriched but related to skin and hair follicles; for example, hair follicle morphogenesis (GO:0031069), hair cycle process (GO:0022405), and positive regulation of hair follicle cell proliferation (GO:0071338). The KEGG pathways associated with target genes of DE lncRNAs between E90d, E120d and Birth of the HF development included the PI3K-Akt, MAPK, FoxO, and cGMP-PKG signaling pathways.

Gene Ontology annotation of DE mRNAs is shown in Supplementary Table S9. The GO terms identified include hair follicle growth terms, such as hair follicle morphogenesis (GO:0031069), hair cycle phase (GO:0044851), hair cycle (GO:0042633), negative regulation of hair cycle (GO:0042636), skin morphogenesis (GO:0043589), and hair cell differentiation (GO:0035315). According to the KEGG pathway analysis of the DE mRNAs, cell adhesion molecules (CAMs) and tight junctions were associated with the growth of skin/hair.

Finally, in the GO enrichment analysis, target genes of DE miRNAs (Supplementary Table S10) were found to be related to aspects of HF development, such as hair cell differentiation (GO:0035315), skin development (GO:0043588), regulation of hair follicle development (GO:0051797), hair cycle process (GO:0022405), hair follicle development and (GO:0001942). According to the KEGG pathway analysis of the DE miRNAs, the cAMP, PI3K-Akt, and AMPK signaling pathways were related to HF development. The detailed information of DE lncRNAs, DE mRNAs, DE miRNAs were listed in Supplementary Table S11.

### Construction of the DE lncRNA–DE miRNA–DE mRNA Network

To increase our understanding of the molecular regulation mechanism of hair follicle growth, we investigated the relationship between lncRNA, miRNA, and mRNA by constructing the DE lncRNA-DE miRNA-DE mRNA network (Supplementary Figures S5, S6). Several interesting lncRNA–miRNA pairs were revealed, such as miR-200a -MSTRG.313133, miR-21/miR-143 and their target lncRNA, MSTRG.172759. The miRNA–mRNA network indicated that several regulatory interactions, such as miR-150-*KRT1*, miR-370-3p and its target gene *KRT32* (gene15074), *KRT35* (gene15075), *VCAN* (gene10153), and *FOXN1* (gene14402), may be important in hair follicle development. Subsequently, according to the principle of ceRNA regulation, we identified target mRNAs and target lncRNAs with the same miRNA binding sites to construct the lncRNA–miRNA–mRNA regulatory network (Supplementary Figure S7). In this model, miRNAs form the center of the network with lncRNA as the bait, and mRNA as the target, suggesting that lncRNA acts as a sponge of miRNA to regulate gene expression. In short, lncRNA and mRNA have the same expression trends that are opposite expression to that of miRNA. In this study, we identified 14 ceRNA pairs that shared 10 miRNAs, such as MSTRG.222885-miR-152-*SPON1*, MSTRG.223165-miR-21-*SOX6*, and MSTRG.308950-miR-103-*IGF2R*, all of which warrant further investigation (Supplementary Table S12).

After further analysis, we found significant differences in the expression levels of miR-21 during the three stages of HF development (Birth-E120d and E120d-E90d), with significantly higher expression at Birth compared with that at E120d, and also at E90d compared with that at E120d. At the same time, we also found significant difference is *SOX6* expression in the E120d-E90d and Birth-E120d groups, with the lowest expression at E90d, and the highest at E120d. Previous studies have shown that miR-21 participates in the growth of animal hair follicles (Zhai et al., 2019), and the SOX family was reported to play a role in cutaneous melanoma (Villani et al., 2017; Noto et al., 2019).

**FIGURE 4:**
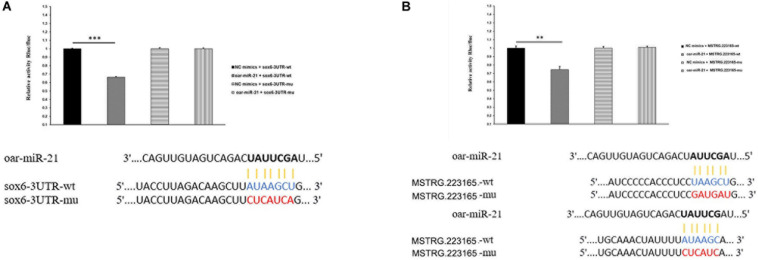
**(A)** miR-21 putative binding sites in *SOX6* 3′-UTR. Pairing schematic of *SOX6* 3′-UTR and miR-21; blue text indicates wild-type sites, and red text indicates mutated sites in the psiCHECK-2 reporter plasmid. The constructs were cotransfected with miR-21 mimics (or negative control) into 293T cells and dual-luciferase assays were performed 48h after transfection. (Data represent mean ± SD, **P < 0.01, ***P < 0.001.) **(B)** miR-21 putative binding sites in MSTRG.223165 3′-UTR. Pairing schematic of MSTRG.223165 3′-UTR and miR-21; blue text indicates wild-type sites, and red text indicates mutated sites in the psiCHECK-2 reporter plasmid. The constructs were cotransfected with miR-21 mimics (or negative control) into 293T cells and dual-luciferase assays were performed 48 h after transfection. (Data represent mean ± SD, **P < 0.01, ***P < 0.001.)

From the results of our study, MSTRG.223165 was identified as a ceRNA for miR-21, which targets *SOX6*. Therefore, we speculated that MSTRG.223165-miR-21-*SOX6* plays a role in the growth of sheep wool follicles. Dual-luciferase assays showed that miR-21 reduced luciferase activity by binding to target sites on MSTRG.223165 and the *SOX6* 3′UTR (Figure 4). These interactions between lncRNA, miRNA, and mRNA indicate a potential regulatory mechanism in skin development and AFWS hair follicle growth.

## Discussion

In many mammals, hair follicles undergo a variety of morphological changes at different times, with certain regularities that exhibit periodicity (anagen, catagen, telogen). Hair growth has been studied in many animal models, including mice (Hansen et al., 1984; Slominski et al., 1994; Kwack et al., 2019), American mink (Zhang et al., 2019a), rabbit (Zhao et al., 2019), yak (Song et al., 2019), and goat (Meyer et al., 1997). Following development from stem cells, hair follicles undergo rapid and cyclical growth, and are surrounded by dermal fibroblasts. During the catagen stage, HFs are surrounded by interfollicular dermal fibroblasts, and the hair bulb starts to shrink. Finally, HFs enter the telogen stage, the hair shaft stops growing, and that hair falls out (Stenn and Paus, 2001). Although many animals have similar hair growth patterns, they may be affected by environmental factors, such as light, season, and temperature (Sun et al., 2019; Zhang et al., 2020). In mammals, SF produce fine hair, which determines wool quality. HE staining showed that PF grew at E90d, while SF were smaller. PF and SF were separated at E120d, and SF matured at Birth, this model supports our research.

In recent years, studies have shown that ncRNAs play an important role in biological processes (Suh, 2018; Tosar et al., 2018), including hair follicle growth cycle (Fu et al., 2014). We used RNA-sequencing technology to identify the lncRNA, mRNA and miRNA molecules expressed in sheep skin during different HF development across two fetal (E90d, E120d) and one postnatal stages (Birth). We detected 461 lncRNAs, 1,009 mRNAs and 106 miRNAs, which were differentially expressed in all comparison groups (Birth-E90, Birth-E120d, and E120d-E90d). We also investigated the expression patterns and functions of lncRNAs, mRNAs and miRNAs to clarify their interactions and relationships with sheep HF development. Several lncRNAs related to hair follicle growth have been reported, such as lnc-000133 and lncRNA-HOTAIR, which participate in hair follicle growth in Cashmere goats (Jiao et al., 2019; Zheng et al., 2019), although the specific functions of lncRNA in hair follicle development remain to be fully elucidated. Studies have shown that mRNAs and miRNAs, such as *FZD4*, *FZD9*, *DKK1*, *BAMBI*, miR-143, and the miR-200 family, play roles in hair follicle development. In accordance with a previous report, we found that *FZD4*, *FZD9*, *DKK1*, and *BAMBI* were differentially expressed during hair follicle development in fine wool sheep (Chen, 2018). miR-143 was detected in our study and has also been reported in association with the HF growth of HU sheep (Huttenhofer et al., 2005). Reports have indicated that the miR-200 family may be a key miRNA in hair follicle development (Hoefert et al., 2018; Liu et al., 2018); thus, we speculate that miR-200b, which was identified in our study, may play a role in the growth of HF of AFWS. Our results were consistent with these reports, provide guidance for our research.

In this study, we identified 493 *cis* and 2,415 *trans* targets in 461 differentially expressed lncRNAs, 1,009 DE mRNAs, and 3,738 target genes for 111 differentially expressed miRNAs. GO annotation and KEGG pathway analysis performed to explore the potential functions and underlying mechanism of these differentially expressed lncRNAs, mRNAs, and miRNAs. GO has a total of three ontologies that describe the molecular function of the gene (MF), the location of the cell (CC), and the biological process involved (BP). KEGG is a database that can be used to decipher the genome by predicting the role of cellular activities in organisms. Our analysis showed that multiple GO terms and signaling pathways form complex mechanisms that regulate the growth of hair follicles. These GO terms included hair follicle development (GO:0001942), hair cycle (GO:0042633), and regulation of hair cycle (GO:0042634).

KEGG pathways include the FoxO, cGMP-PKG, cAMP, PI3K-Akt, and AMPK signaling pathways. Previous studies have shown that these pathways participate in, or regulate the growth process of wool follicles (Lv et al., 2017; Jiankui et al., 2019; Ranran et al., 2020). It was found that *BRAF* is enriched in CAMP and FoxO signaling pathways, and *PIK3R3* is enriched in the CAMP, JAK-STAT, and AMP signaling pathways. In this study, we found that *BRAF* and *PIK3R3* were significantly differentially expressed and downregulated in E120d-E90d. *BRAF* inhibitors are involved in the development of hair curls and have been used treat hair loss as well as in hair care products (Keating and Dasanu, 2017). In mice with spontaneous fur mutations, researchers found that *PIK3R3* may affect cell cycle, immune response and skin development during mouse growth (Ji et al., 2017). We also found that MSTRG.172760, MSTRG.421324, MSTRG.482102, MSTRG.535526, MSTRG.610044, MSTRG.653547, MSTRG.722949, MSTRG.730613 and gene3930 all target gene360 (*PIK3R3*). MSTRG.173186, MSTRG.225489, and MSTRG.617489 target gene8419 (*BRAF*), suggesting that these lncRNAs regulate hair follicle growth through gene360 (*PIK3R3*) and gene8419 (BRAF), which are therefore implicated as candidate genes for hair follicle growth.

miRNA–lncRNA and miRNA–mRNA networks provide clues for analyzing gene regulation. In our study, we found several interesting networks, such as miR-200a-MSTRG.313133, miR-21/miR-143 and their target lncRNA MSTRG.172759. Among them, the miRNAs were associated with hair growth, and their target gene lncRNAs were expressed at high levels with differential expression patterns during the growth. It has been reported that the miR-200 family regulates cell adhesion in the hair germ cells and influence morphogenesis of the hair follicle (Hoefert et al., 2018). miR-21 may affect hair follicle growth by regulating its target genes (Zhai et al., 2019), and miR-143 is a candidate gene in the regulation of hair follicle growth in Hu sheep (Gao et al., 2017). Thus, this network may play a role in regulating the growth of hair follicles. In addition, miRNA–mRNA networks, such as miR-150-KRT1, miR-370-3p and its target gene *KRT32* (gene15074), *KRT35* (gene15075), *VCAN* (gene10153), *FOXN1* (gene14402) and their target genes have been studied in relation to hair follicle or skin growth. miR-150 participates in the regulation the formation of hair follicle melanoma (Wang et al., 2019). miR-370-3p acts as a target of circRNA_NEK6 and plays a role in proliferation of thyroid carcinoma (Chen et al., 2018). The KRT family is related to the degree of curl and shape of horsehair (Morgenthaler et al., 2017). *VCAN* is a marker gene for hair papilla cells, and its growth rate is not affected by external light intensity (Jampa-Ngern et al., 2017). *FOXN1* may affect the growth of secondary hair follicles in Cashmere goats (Zhang et al., 2019c).

The mechanism of ceRNA regulation has been extensively researched in various diseases (Tang et al., 2019; Tao et al., 2019). Recently, TMPO-AS1 has been identified as a competitive endogenous RNA that promotes tumorigenesis of osteosarcoma by regulating the miR-199a-5p/*WNT7B* axis, which provides a potential therapeutic target for osteosarcoma patients (Cui and Zhao, 2019). Studies of The Cancer Genome Atlas (TCGA) database showed that lncRNA MATN1-AS1 promotes the progression of gliomas by regulating the miR-200b/c/429-*CHD1* axis, suggesting that MATN1-AS1 may be a therapeutic target for glioma (Zhu et al., 2020). In a study of ncRNA and RNA in the cashmere goat hair follicle cycle, Wang et al. (2017) constructed ceRNA networks by RNA-seq and bioinformatics analysis. Based on the results, three ceRNA networks, miR-221-5p-lnc_000679-*WNT3*, were selected as candidate ceRNA networks that may be involved in the regulation of hair follicle growth. Zhang first studied the regulatory relationship of lncRNA–mRNA in the development of fine wool follicles (Yangfan et al., 2018), which provided a reference for our research. Our experiment is the first study to provide RNA-seq data for hair follicle development from the fetal stage to birth in AFWS, and also the first study on the lncRNA–miRNA–mRNA regulatory network in different development stages of AFWS. The ceRNA results show that some lncRNA–mRNA interact with multiple different miRNAs, and one miRNA can also target multiple mRNAs at the same time. This phenomenon indicates that miRNA occupies a central position in the ceRNA network, linking lncRNA and mRNA. Collation of the target mRNA and target lncRNA data with the same miRNA binding site revealed that interactions such as MSTRG.222885-miR-152-*SPON1*, MSTRG.223165-miR-21-*SOX6*, and MSTRG.308950 -miR-103 -*IGF2R* are involved in the ceRNA network. It was found that *SPON1* regulated the growth, differentiation and morphogenesis of hair follicles via Wnt, BMP and other signaling pathways (Muhammad et al., 2019). It has been reported that *IGF2R* as a target gene of miR-211 activates the MAPK signaling pathway and thereby inhibits the formation of skin melanoma (Dror et al., 2016). MAPK is also a signaling pathway for hair follicle growth and development and *IGF2R* may also affect hair color and hair follicle growth through this signal pathway (Xiao et al., 2019).

In this study, we mainly studied the network of MSTRG.223165-miR-21-*SOX6*, in which MSTRG.223165 sponges miR-21, which targets *SOX6*; thus, this provides important information about the growth of AFWS hair follicles. It has been reported thatmiRNA-21 is an important downstream component of BMP signaling and plays a significant role in skin development (Ahmed et al., 2011). Studies of super merino and small-tailed Han sheep showed that *CNKSR2*, *KLF3* and *TNPO1* are the target genes of miR-21, and may therefore be involved in the function of miR-21 in hair follicle development (Zhai et al., 2019). The Sox family is expressed in early hair follicle development, and Sox13, which is essential for epidermal and adnexal development, can be used as a useful marker of early hair follicle development (Noto et al., 2019). *SOX2* and *SOX18* are critical for determining the type of hair follicle, and *Sox18* regulates the normal differentiation of dermal papillae of all hair types (Villani et al., 2017). During the differentiation of neural stem cells, miR-21 expression was increased, and reduced SOX protein expression by binding to *SOX2*, thereby promoting differentiation (Ni et al., 2014). In our analysis of DE lncRNA-DE miRNA-DE mRNA, we found that MSTRG.223165, miR-21 and *SOX6* were correlated, suggesting that MSTRG.223165 regulated the expression of Sox6. Thus, we hypothesize that MSTRG.223165 functions as a regulatory gene in the growth of hair follicles, although the molecular regulation mechanism remains to be clarified.

## Conclusion

In this study, we investigated the expression of lncRNAs, mRNAs, and miRNAs in the skin during different developmental stages in AFWS. GO annotation and KEGG pathway analysis were used to identify the candidate lncRNAs, mRNAs, and miRNAs in the developmental stages of the wool follicle growth. We also constructed the ceRNA networks of MSTRG.223165 -miR-21- *SOX6* and dual-luciferase assays were used to verify the target relationships in this network to investigate the factors that affect wool follicle growth. These results provide the foundation for further exploration of the molecular mechanism underlying the regulation of wool growth.

## Data Availability Statement

Our data is being uploaded to the SRA database, the lncRNA-Seq and mRNA-Seq data was submitted to the SRA database under accession number SRP240733. miRNA-Seq data was submitted to the SRA database under accession number SRP253232.

## Ethics Statement

All experimental and surgical procedures involved in this study followed the “Guidelines for Experimental Animals” of the Ministry of Science and Technology (Beijing, China). Operations and Animal Care were sustained by the experimental animal ethics committee of Qingdao Agricultural University.

## Author Contributions

RZ, JL, and NL conceived and designed this study. RZ, NL, LrL, and FY participated in sample collection. RZ, JL, and LlL performed the experiments. RZ, YW, FY, and HL analyzed the data and prepared the figures and tables. RZ, NL, and JH wrote the manuscript. All authors reviewed and approved the final manuscript.

## Conflict of Interest

The authors declare that the research was conducted in the absence of any commercial or financial relationships that could be construed as a potential conflict of interest.

## Footnotes

1 www.mirbase.org

## Supplementary Material

FIGURE S1 Comparative analysis of lncRNAs and mRNAs. **(A)** Distribution of exon numbers. **(B)** Length distribution. **(C)** Expression levels. **(D)** JS score density distribution.

FIGURE S2 Identification of differentially expressed lncRNAs during three developmental stages of AFWS. **(A–C)** Volcano plots showing the differentially expressed lncRNAs in pairwise comparison groups (upregulated or downregulated). **(D)** Venn diagram showing the number of overlapping differentially expressed lncRNAs in compared groups. **(E)** Heatmap of differentially expressed lncRNAs; yellow and blue indicate high and expression levels, respectively.

FIGURE S3 Identification of differentially expressed mRNA during three developmental stages of AFWS. **(A–C)** Volcano plots showing the differentially expressed mRNAs in pairwise comparison groups (upregulated or downregulated). **(D)** Venn diagram showing the number of overlapping differentially expressed mRNAs in compared groups. **(E)** Heatmap of differentially expressed mRNAs; yellow and blue indicate high and expression levels, respectively.

FIGURE S4 Identification of differentially expressed miRNA during three developmental stages of AFWS. **(A–C)** Volcano plots showing the differentially expressed miRNAs in pairwise comparison groups (upregulated or downregulated). **(D)** Venn diagram showing the number of overlapping differentially expressed miRNAs in compared groups. **(E)** Heatmap of differentially expressed miRNAs; yellow and blue indicate high and expression levels, respectively.

FIGURE S5 Interactions between miRNA and mRNAs in AFWS wool follicles. **(A)** miRNA–mRNA network between birth and E90d. **(B)** miRNA–mRNA network between birth and E120d. **(C)** miRNA–mRNA network between E120d and E90d.

FIGURE S6 Interactions between lncRNAs and miRNAs in AFWS wool follicles. **(A)** lncRNA–miRNA network between birth and E90d. **(B)** lncRNA–miRNA network between birth and E120d. **(C)** lncRNA–miRNA network between E120d and E90d.

FIGURE S7 Interactions of lncRNA–miRNA–mRNA in AFWS wool follicles. **(A)** lncRNA–miRNA–mRNA network between birth and E90d. **(B)** lncRNA–miRNA–mRNA network between birth and E120d. **(C)** lncRNA–miRNA–mRNA network between E120d and E90d.

TABLE S1 Detailed information of primers used for qRT-PCR analysis.

TABLE S2 Target mRNA prediction (*cis* and *trans*) of lncRNA.

TABLE S3 Annotated mature miRNAs and precursors.

TABLE S4 Novel mature miRNA sequence and precursor sequence.

TABLE S5 Identification of differentially expressed lncRNAs during three developmental stages of AFWS. **(A–C)** Volcano plots showing the differentially expressed lncRNAs in pairwise comparison groups (upregulated or downregulated). **(D)** Venn diagram showing the number of overlapping differentially expressed lncRNAs in compared groups. **(E)** Heatmap of differentially expressed lncRNAs; yellow and blue indicate high and expression levels, respectively.

TABLE S6 Identification of differentially expressed mRNA during three developmental stages of AFWS. **(A–C)** Volcano plots showing the differentially expressed mRNAs in pairwise comparison groups (upregulated or downregulated). **(D)** Venn diagram showing the number of overlapping differentially expressed mRNAs in compared groups. **(E)** Heatmap of differentially expressed mRNAs; yellow and blue indicate high and expression levels, respectively.

TABLE S7 Identification of differentially expressed miRNA during three developmental stages of AFWS. **(A–C)** Volcano plots showing the differentially expressed miRNAs in pairwise comparison groups (upregulated or downregulated). **(D)** Venn diagram showing the number of overlapping differentially expressed miRNAs in compared groups. **(E)** Heatmap of differentially expressed miRNAs; yellow and blue indicate high and expression levels, respectively.

TABLE S8 Gene ontology annotation of differentially expressed lncRNAs between E90d, E120d, and birth.

TABLE S9 Gene ontology annotation of differentially expressed mRNAs between E90d, E120d, and birth.

TABLE S10 Gene ontology annotation of differentially expressed miRNAs between E90d, E120d, and birth.

TABLE S11 Detailed results of KEGG pathway analysis of lncRNAs, mRNAs, and miRNAs.

TABLE S12 Fourteen pairs of ceRNAs consistent with the ceRNA regulation mechanism.
