# Supplementary figures and images for: Transcriptomic Analysis Reveals the Involvement of lncRNA–miRNA–mRNA Networks in Hair Follicle Induction in Aohan Fine Wool Sheep Skin
Source: Front Genet. 2020 Jun 9;11:590. doi: 10.3389/fgene.2020.00590 (PMC7528302; doi:10.3389/fgene.2020.00590)

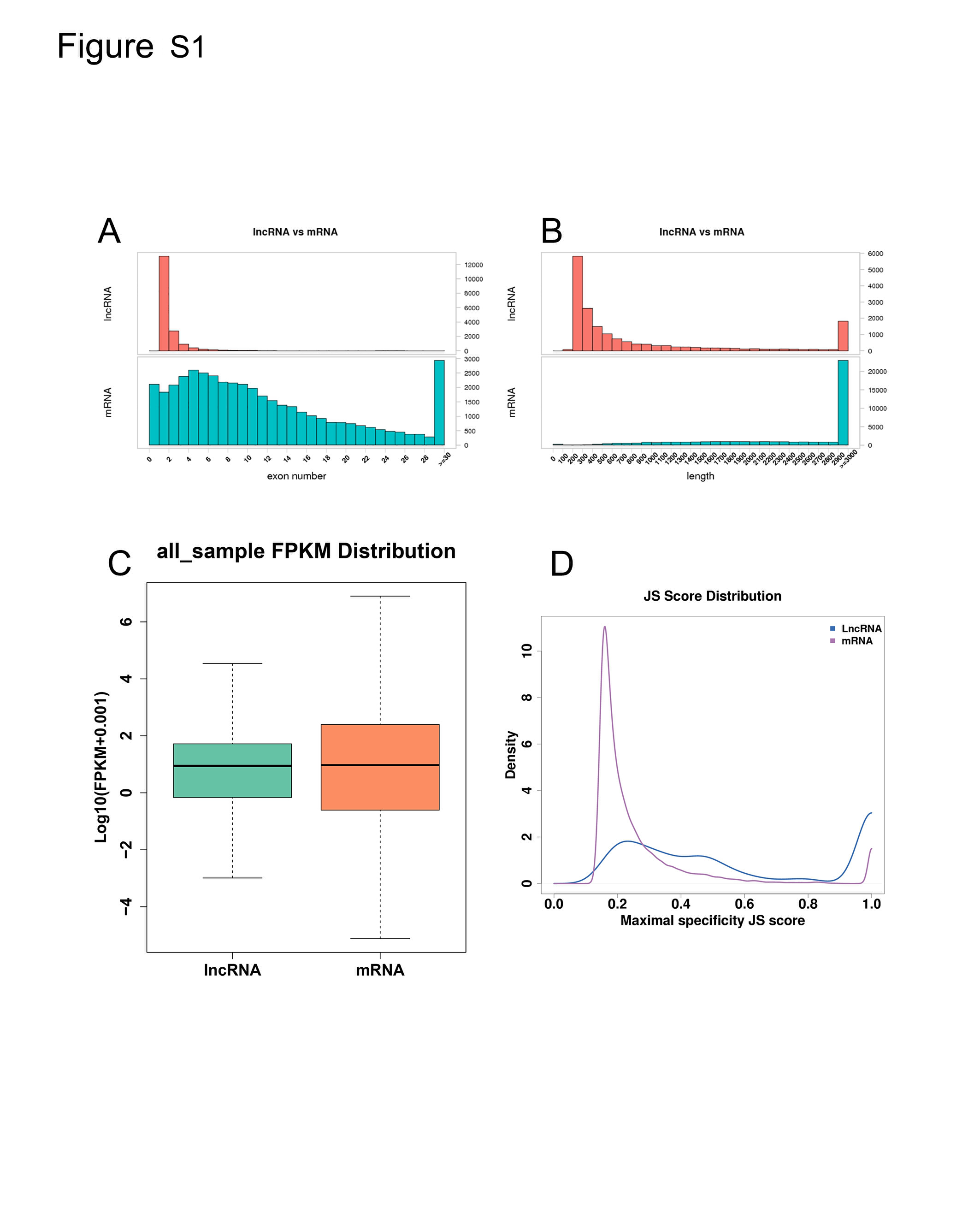

Supplement: FIGURE S1 — Comparative analysis of lncRNAs and mRNAs. (A) Distribution of exon numbers. (B) Length distribution. (C) Expression levels. (D) JS score density distribution. [file Image_1.TIF]

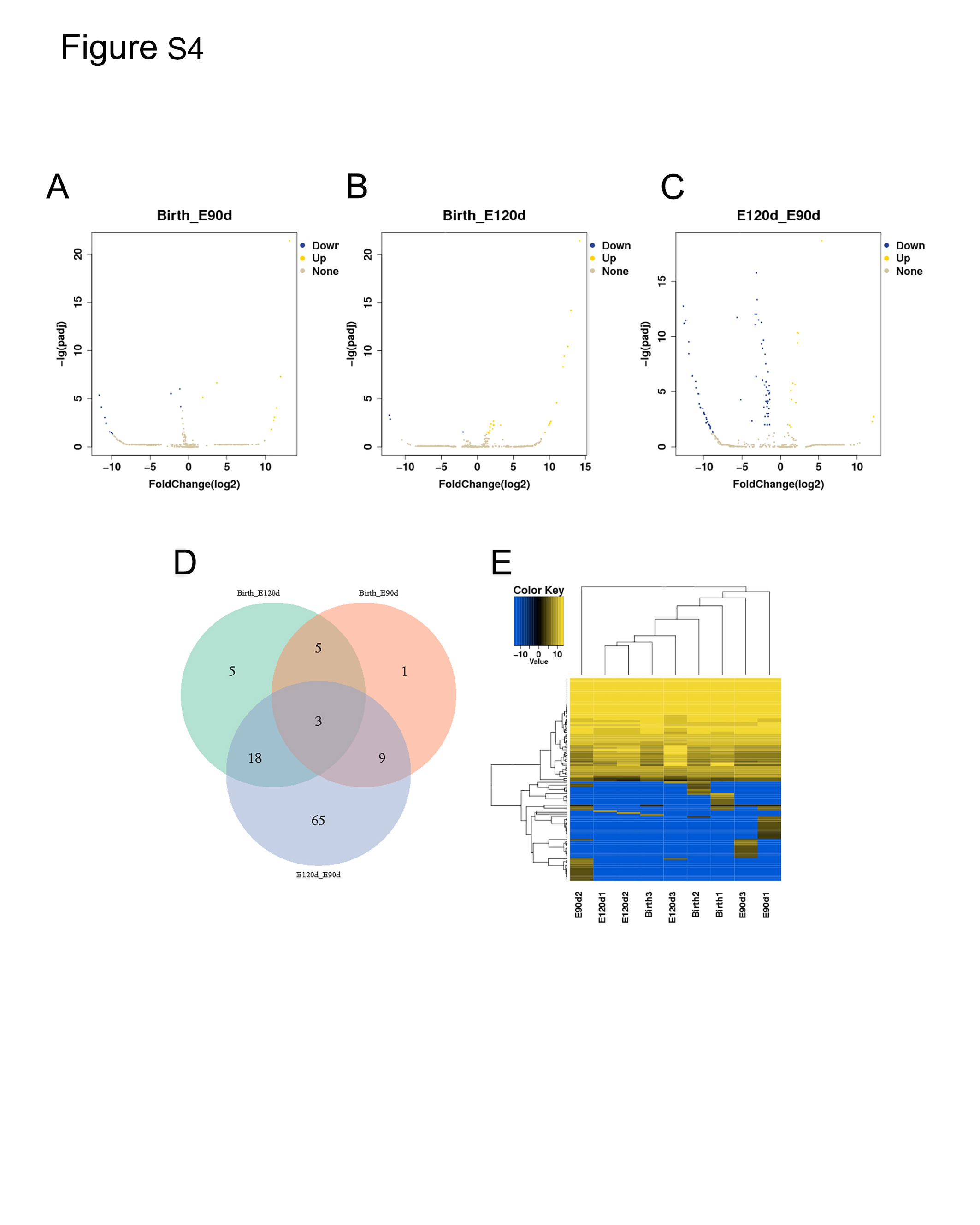

Supplement: FIGURE S4 — Identification of differentially expressed miRNA during three developmental stages of AFWS. (A–C) Volcano plots showing the differentially expressed miRNAs in pairwise comparison groups (upregulated or downregulated). (D) Venn diagram showing the number of overlapping differentially expressed miRNAs in compared groups. (E) Heatmap of differentially expressed miRNAs; yellow and blue indicate high and expression levels, respectively. [file Image_4.TIF]

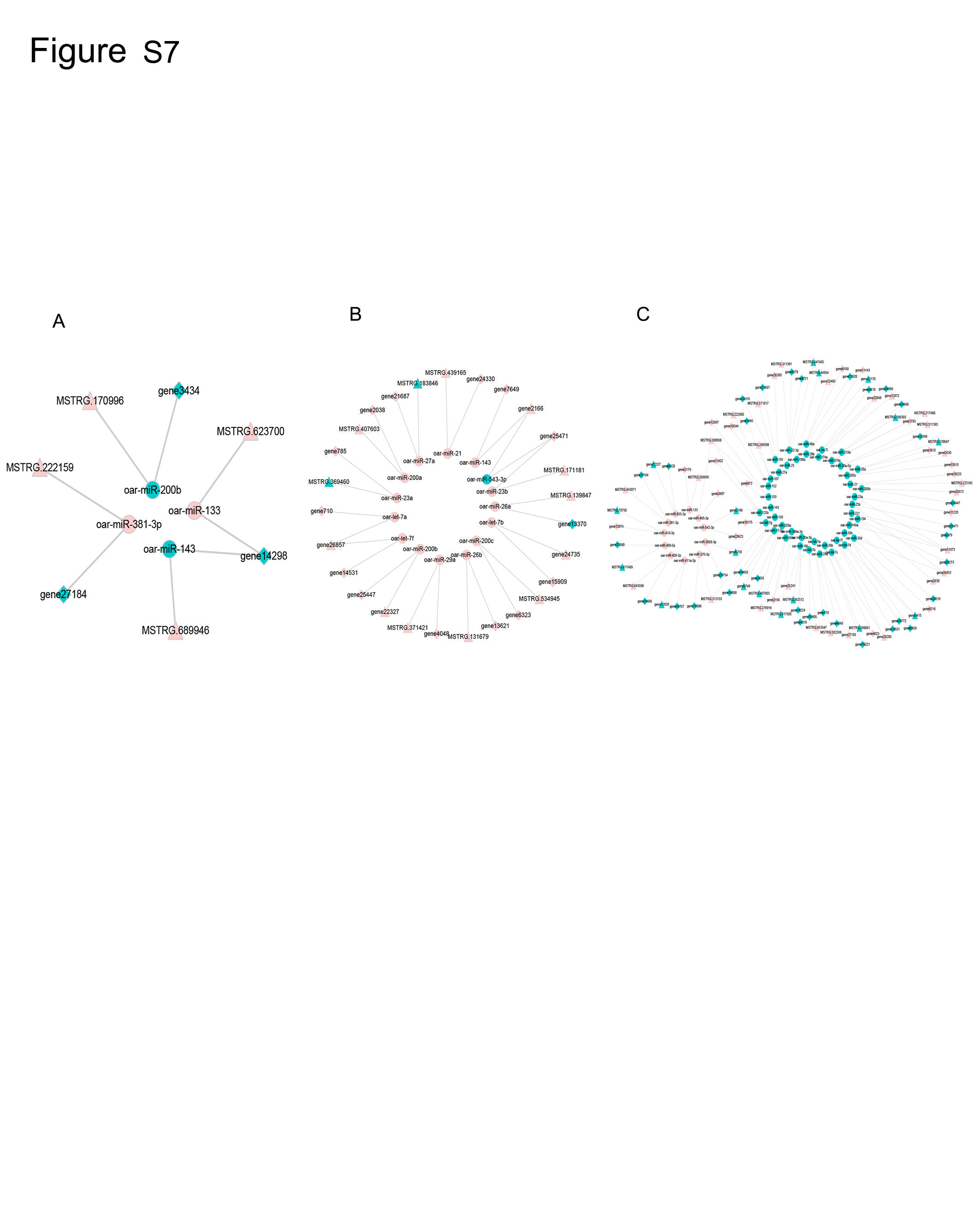

Supplement: FIGURE S7 — Interactions of lncRNA–miRNA–mRNA in AFWS wool follicles. (A) lncRNA–miRNA–mRNA network between birth and E90d. (B) lncRNA–miRNA–mRNA network between birth and E120d. (C) lncRNA–miRNA–mRNA network between E120d and E90d. [file Image_7.TIF]
